# Finite Element Simulation of Opening Angle Response of Porcine Aortas Using Layer Specific GAG Distributions in One and Two Layered Solid Matrices

**DOI:** 10.1007/s13239-024-00754-x

**Published:** 2024-10-02

**Authors:** Noor M. Ghadie, Jean-Philippe St-Pierre, Michel R. Labrosse

**Affiliations:** 1 Department of Mechanical Engineering, University of Ottawa, Ottawa, ON K1N 6N5 Canada; 2 Department of Chemical and Biological Engineering, University of Ottawa, Ottawa, ON K1N 6N5 Canada; 3 Department of Cardiac Surgery, University of Ottawa Heart Institute, Ottawa, ON K1Y 4W7 Canada

**Keywords:** Donnan swelling, Glycosaminoglycan, Layer-specific modeling, Opening angle, Residual stress

## Abstract

**Purpose:**

Recent studies have identified an effect of glycosaminoglycans (GAG) on residual stresses in the aorta, underscoring the need to better understand their biomechanical roles.

**Methods:**

Aortic ring models for each of the ascending, arch and descending thoracic regions of the porcine thoracic aorta were created in FEBioStudio, using a framework that incorporates the Donnan osmotic swelling in a porous solid matrix. The distribution of fixed charge densities (FCD) through the thickness of the tissue was prescribed as calculated from experimentally quantified sulfated GAG mural distributions. Material parameters for the solid matrix, modeled using a Holmes–Mow constitutive law, were optimized using data from biaxial tensile tests. In addition to modelling the solid matrix as one layer, two layers were considered to capture the differences between the intima-media and the adventitia, for which various stiffness ratios were explored.

**Results:**

As the stiffness of the adventitia with respect to that of the media increased, the simulated opening angle increased. The opening angle also decreased from the ascending to the descending thoracic region in both one- and two-layered solid matrices models. The simulated results were compared against the experimental contribution of GAG to the opening angle, as previously quantified via enzymatic GAG-depletion. When using one layer for the solid matrix, the errors between the simulated opening angles and the experimental contribution of GAG to the opening angle were respectively 28%, 15% and 23% in the ascending, arch and descending thoracic regions. When using two layers for the solid matrix, the smallest errors in the ascending and arch regions were 21% and 5% when the intima-media was modelled as 10 times stiffer, and as twice stiffer than the adventitia, respectively, and 23% in the descending thoracic regions when the intima-media and adventitia shared similar mechanical properties.

**Conclusions:**

Overall, this study demonstrates that GAG partially contribute to circumferential residual stress, and that GAG swelling is one of several regulators of the opening angle. The minor discrepancies between simulated and experimental opening angles imply that the contribution of GAG extends beyond mere swelling, aligning with previous experimental indications of their interaction with ECM fibers in determining the opening angle.

**Supplementary Information:**

The online version contains supplementary material available at 10.1007/s13239-024-00754-x.

## Introduction

The existence of circumferential residual stresses in blood vessels was revealed in the 1980’s, when Vaishnav and Vossoughi [1], and Chuong and Fung [2] showed that a radial cut through the vessel’s wall in the unloaded state causes it to spring open. The open state of a tubular shaped aortic segment can be characterized by the opening angle. In the case of the aorta, knowledge of residual stresses is important for several reasons. First, their relief, such as approximated by a radial cut, provides an unloaded reference geometry from which the mechanical stresses and strains can be calculated [2, 3]. Secondly, residual stresses are thought to ensure a homogeneous distribution of the *in vivo* mechanical stresses across the aortic wall [2, 4]. Finally, understanding the mechanisms causing alterations in residual stresses is of significant interest as it may provide insights into aortic pathogenesis [5, 6]. It is therefore widely accepted that inclusion of residual stresses in computational models is of paramount importance to obtain an accurate assessment of the *in vivo* mechanical stress field of the tissue. Indeed, tissue ruptures will occur when the *in vivo* mechanical stresses exceed the strength of the aorta.

The development of computational models also requires the use of constitutive equations to describe the mechanical response of the tissue to loading. *In silico* models may consider one or multiple layers of the aortic wall [7–9], whereby using multiple layers allows one to account for the distinct mechanical properties that the different layers of the aortic wall may exhibit [10, 11]. The main layers of the aortic wall are the intima (the innermost layer from the lumen), the media (the middle layer) and the adventitia (the outermost layer). These layers occupy different proportions of the aortic wall at different locations along its tree [12], and contain a mixture of cells along with a well organized extracellular matrix (ECM). The ECM of the aortic wall is mainly comprised of elastin, collagen and glycosaminoglycans (GAG). The content and organization of the ECM components vary through the aortic wall thickness and based on location along the aortic tree. For instance, the collagen content increases from the intima to the adventitia and the GAG content decreases, while elastin exists in its highest levels in the media [13]. Furthermore, while the collagen content increases in the aortic tree as one moves away from the heart [13, 14], the GAG [13, 14] and the elastin [14, 15] contents have been shown to decrease. In addition, these ECM constituents influence the mechanical properties of the aorta, such as stiffness [16–19], and residual stresses [4, 19, 20].

Different methods for incorporating residual stresses in computational models have been established. The most common methods have focused on either setting a known opening angle [7, 21, 22], or incorporating ad-hoc local prestretches [23–25]. On the one hand, knowledge of the opening angle allows one to determine the residual stress field by putting the opened sector back into a closed tubular shape, or in other words, by mapping the open unstressed configuration to the closed unloaded state. On the other hand, the residual stress field can be created via the inclusion of *in situ* prestretches, which have been associated with the fibrous components of the ECM, namely elastin and collagen.

The seminal work by Azeloglu et al. demonstrated the possibility of recovering the opening angle computationally via the inclusion of fixed charge densities (FCD) to simulate the Donnan swelling effect of GAG [26]. However, *ex vivo* experiments performed by our group recently showed that, although the GAG content and gradient in the aorta strongly correlate with its opening angle [13] and that the enzymatic removal of GAG yields a significant reduction in the opening angle [19], this reduction represented approximately 33% of the total angle, with variations as a function of position along the aortic tree [19]. This suggested that while GAG play a significant role in regulating circumferential residual stress, particularly considering its relatively low content in the tissue, the inclusion of FCD in a computational model of the aortic ring is not expected to result in the full recovery of experimentally determined opening angles on its own.

Due to the lack of comprehensive species-specific data detailing aortic composition, organization and mechanical properties, the computational model in Ref. [26] incorporated composite information sourced from the characterization of tissues from different species and animals with different levels of maturity. Specifically, the FCD mural distribution prescribed in the rodent aortic ring model was first estimated based on published data by Porterfield et al. on Leghorn chickens [27], which were then compared against estimates from histological observation along the aortic wall and the average GAG content from the full thickness of rodent aortas. Roccabianca et al. further simulated the role of GAG pooling in human thoracic aortic dissection [28], as well as their mechanosensing roles in murine carotid arteries [29]. In both studies, the normal GAG levels used were also based on results from Porterfield et al. These foundational efforts to elucidate the roles of GAG in the mechanical behaviour of the aorta highlighted the need to increase experimental information on GAG including its distribution in the tissue, in order to advance our knowledge on their mechanical roles. However, no subsequent efforts have been made to evaluate the feasibility of using prescribed FCD distributions in the aortic wall to recover the unloaded but residually stressed configuration of an aortic ring.

Our previous efforts toward understanding the mechanical roles of GAG in the porcine thoracic aorta generated refined data on the mural distribution of sulfated GAG (sGAG) in the ascending, arch and descending thoracic regions of the aorta, as our experimental approach allowed us to quantify sGAG levels in thin slices throughout the aortic wall. This study also revealed that GAG exist in the ascending region in significantly higher quantities compared to the arch and descending thoracic regions [13]. Porterfield et al. also showed that the GAG content decreases with increasing age of Leghorn chickens [27], further highlighting the differences in GAG quantities between species, age, and anatomical location. In light of our recent detailed data on mural GAG distributions in the different regions of the porcine thoracic aorta [13], and given the mechanical data that we obtained from biaxial tensile tests on samples excised from the same regions [18], in the present study, we aimed to use these previously published experimental parameters obtained from porcine animals aged 5- to 6-months (1) to corroborate our prior experimental findings, as discussed previously in the introduction, on the contribution of GAG to residual stress through in silico modelling, (2) to evaluate the reliability of using the Donnan osmotic swelling mathematical law to illustrate the contribution of GAG to the opening angle computationally, and (3) to evaluate the opening angle response in models using one layer for the solid matrix versus two layers, in order to illustrate the possible impact of differences in mechanical properties between the intima bundled with the media, and the adventitia.

## Methods

### General Framework

In this work, we used a mixture-based finite element model in FEBioStudio Version 1.7 [30] (https://febio.org/), as implemented by Azeloglu et al. [26]. The mixture combined a porous solid matrix and a Donnan equilibrium swelling material, which describes the presence of FCD illustrating the negative charges of GAG, with a fluid phase. The Cauchy stress tensor ${\varvec{\sigma}}$ for the mixture (Eq. 1) combines the fluid pressure $\pi$ and the elastic stress ${{\varvec{\sigma}}}^{{\varvec{e}}}$ associated with the solid matrix, as given in Ref. [26]:

1 $${\varvec{\sigma}}=-\pi {\varvec{I}}+{{\varvec{\sigma}}}^{{\varvec{e}}}$$

where $I$ is the identity tensor. While $\pi$ arises from both the ambient pressure (i.e., pressure of the external bath) and the Donnan osmotic pressure, at equilibrium, the ambient pressure is null, and thus, $\pi$ reduces to the Donnan osmotic pressure, expressed as follows:

2 $$\pi =RT\Phi \left(\sqrt{{{(c}^{F})}^{2}+{\left({\overline{c} }^{*}\right)}^{2}}-{\overline{c} }^{*}\right)$$

In Eq. 2, *R* is the universal gas constant, *T* is the temperature, $\Phi$ is the osmotic coefficient and is equal to 1 for an ideal system, ${\overline{c} }^{*}$ is the external bath osmolarity, and ${c}^{F}$ is the proteoglycan FCD in the current configuration and relates to the reference configuration via the equation below:

3 $${c}^{F}=\frac{{\varphi }_{0}^{\omega }}{J-1+{\varphi }_{0}^{\omega }}{c}_{0}^{F}$$

where $J$ is the relative volume and ${\varphi }_{0}^{\omega }$ and ${c}_{0}^{F}$ are the fluid volume fraction and FCD in the reference configuration, respectively.

The general expression for the elastic stress ${{\varvec{\sigma}}}^{{\varvec{e}}}$ for the solid matrix relates to the second Piola-Kirchhof (P–K) stress tensor ${{\varvec{T}}}^{{\varvec{S}}}$ via the expression provided in Eq. 4. Replacing ${{\varvec{T}}}^{{\varvec{S}}}=2 \partial W/\partial {\varvec{C}}$ in Eq. 4 provides the final expression of the stress tensor of the solid matrix, as shown in Eq. 5. In these equations, ${\varvec{F}}$ is the deformation gradient, $W({\varvec{C}})$ is the strain energy density function (details on constitutive formulation is provided in “Solid Matrix” Sect.) and ${\varvec{C}}={{\varvec{F}}}^{{\varvec{T}}}\bullet {\varvec{F}}$ is the right Cauchy-Green deformation tensor [31].

4 $${{\varvec{\sigma}}}^{{\varvec{e}}}=\frac{1}{J}{\varvec{F}}\cdot {{\varvec{T}}}^{{\varvec{S}}}\cdot {{\varvec{F}}}^{{\varvec{T}}}$$

5 $${{\varvec{\sigma}}}^{{\varvec{e}}}=\frac{2}{J}{\varvec{F}}\cdot \frac{\partial W}{\partial {\varvec{C}}}\cdot {{\varvec{F}}}^{{\varvec{T}}}$$

**Fig. 1:**
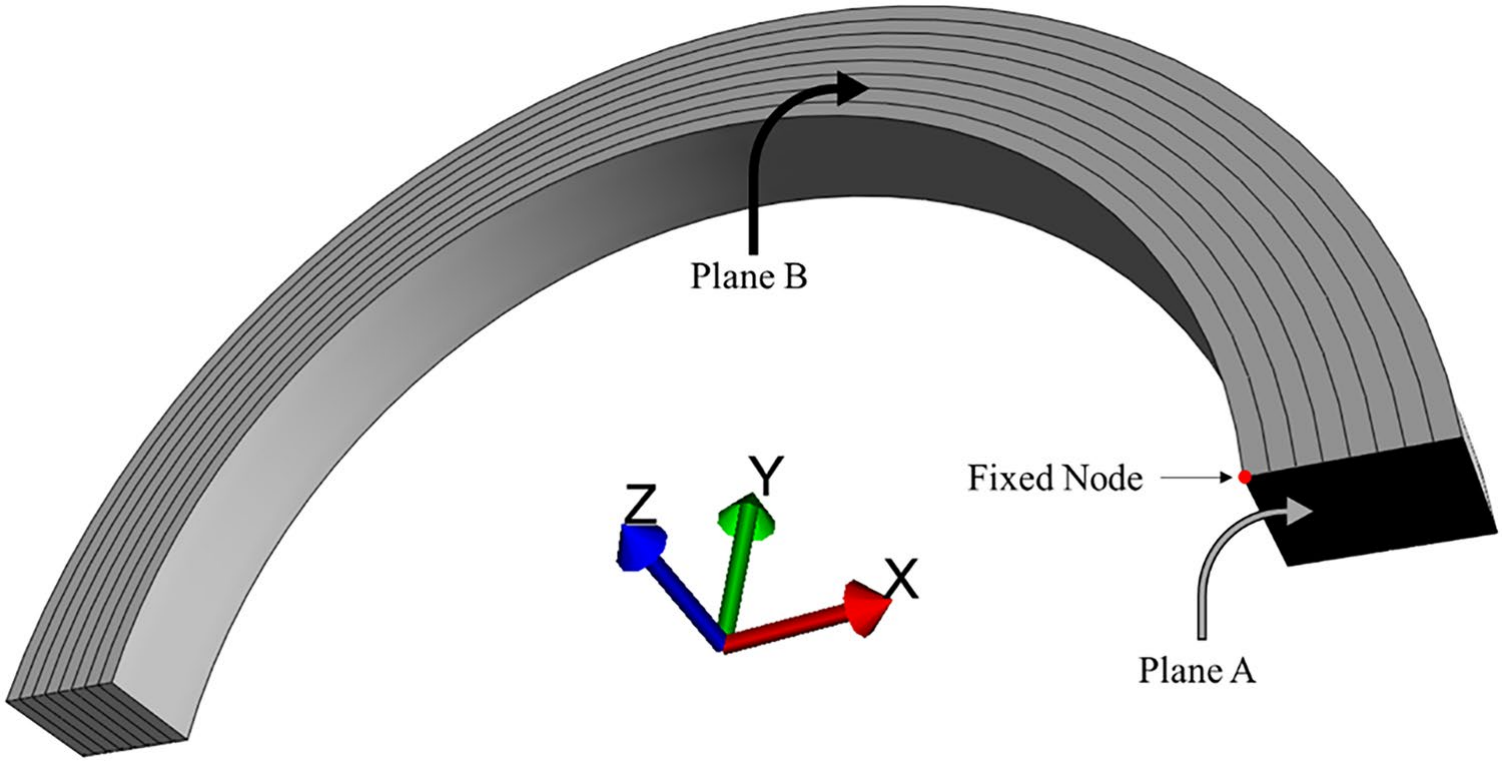
Representative quarter aortic ring geometry from the ascending region displaying boundary conditions. Plane A is fixed in the y-direction, plane B is fixed along the z-direction, and the fixed node is constrained in all directions. One-quarter ring has 8 elements across the aortic wall thickness, 20 elements along the circumference, and 8 elements along its height

This approach was used to model aortic rings from the ascending, aortic arch and descending thoracic regions of porcine aortas from 5- to 6-months old animals harvested in our lab in previous studies [13, 19]. The approach was adapted from [26], in which residual stresses in rodent tissue were studied, and by comparison with which we initially validated the framework in Ref. [32]. Specifically, in the present study, we have refined several parameters included in the model by incorporation of experimental data specific to 5- to 6-months old porcine animals collected by our group. While it has been previously established that the opening angle is sensitive to the external bath osmolarity [26], this study maintained a physiological osmolarity of 300 mosM, in order to ensure that meaningful comparisons could be made with our previously published experimental results, in which the aortic rings were immersed at this same osmolarity to mimic physiological conditions [13, 19]. The rings were meshed using 20-node quadratic hexahedral elements, with 8 elements across the aortic wall thickness, 40 elements along the circumference of the full ring, and 8 elements along the ring height (i.e., aortic slice thickness). Appropriate boundary conditions to represent symmetry, and the radial cut on one side, were imposed to model only one-quarter of a ring (i.e., half height, and half circumference), as shown in Fig. 1. Briefly, planes A and B in Fig. 1 are fixed in the y- and z-directions, respectively, and the “Fixed Node” lying in these two planes is fixed in all directions. Convergence of solutions was verified using a classical mesh sensitivity analysis. Parameters for geometry, FCD distributions and mechanical behaviour of the solid matrix for each of the ascending, aortic arch, and descending thoracic (from in between the third and fifth intercostal arteries) rings were acquired from previous experiments carried out in our lab [13, 18], as described next.

### Geometry

The model parameters described in this section were averaged from a total of 15 rings per region from 5 porcine aortas that we investigated in Ref. [13]. The outer diameters and thicknesses of the computer ring models for each of the ascending, aortic arch and descending thoracic regions are summarized in Table 1, and a ring height of 4.5 mm was used for all 3 models.

**Table 1:** Outer diameter and thickness (mm) used in the computer models of rings from the ascending, arch and descending thoracic regions averaged from 15 porcine aortic rings in Ref. [13]

Region	Outer diameter (mm)	Mural thickness (mm)
Ascending	25.5	2.68
Arch	22.0	2.56
Descending thoracic	17.6	1.82

### FCD Distribution

The average sGAG masses were interpolated across 8 equally sized domains through the wall thickness (corresponding to as many elements in the computer model) using quantitative normalized sGAG measurements published in Ref. [13] (cf. Fig. 3b), where the intramural sGAG distribution in the ascending, aortic arch, and descending thoracic regions of porcine aorta was measured using a DMMB spectrophotometric assay on slices cryo-sectioned from the aortic wall. This allowed us to compute the FCD distributions in each of the ascending, aortic arch, and descending thoracic regions (see Table 2) using the formula [26]:

**Table 2:** Fixed charge densities [mEq/L] in aortic wall domains for each of the ascending, arch, and descending thoracic ring models, calculated from sGAG measurements published in Ref. [13]

Mural position	Ascending	Arch	Descending thoracic
1	− 35.3	− 25.3	− 19.4
2	− 32.1	− 20.5	− 16.8
3	− 29.6	− 17.4	− 17.8
4	− 25.8	− 16.2	− 17.9
5	− 21.7	− 14.9	− 15.7
6	− 19.9	− 14.2	− 14.3
7	− 18.1	− 12.9	− 12.7
8	− 15.0	− 9.7	− 9.0

6 $${c}^{F}=\frac{GAG\, charge\, number}{GAG\, molecular\, weight}\times \frac{GAG\, mass}{water\, volume}$$

Consistent with [26], each chondroitin sulfate isomer was assumed to have a molecular weight of 513 g/mol with two negative charges. The water content was assumed to be 70%.

### Aortic Layers

**Fig. 2:**
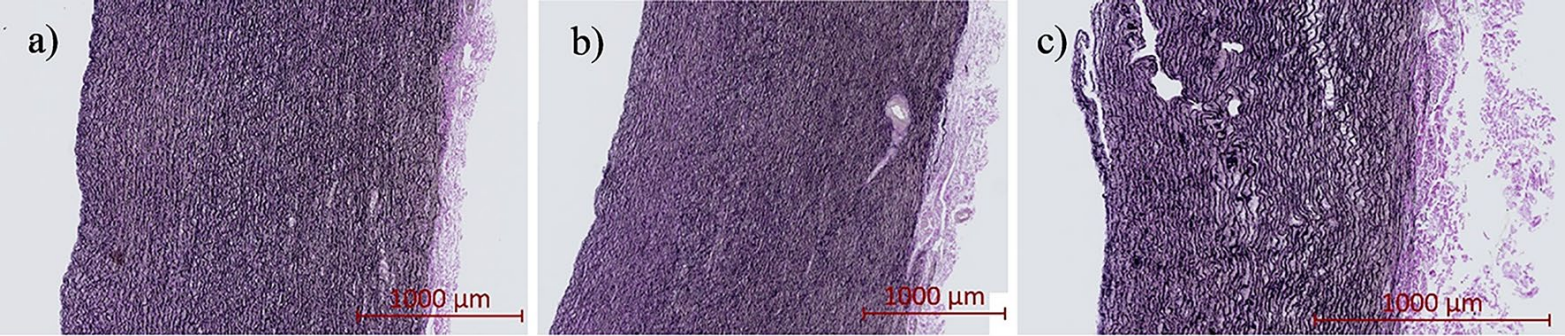
Representative histological sections of aortic tissues from **a** the ascending, **b** aortic arch, and **c** descending thoracic aorta, stained with Verhoeff’s-VanGieson’s. Intimal layers are aligned with the left side of each panel

The proportions of the intima-media and adventitia in each of the investigated anatomical regions were obtained from samples procured from 4 porcine aortas. Samples were excised from each of the ascending, arch, and descending thoracic regions and were fixed in 10% formalin solution for 72 h, then transferred to 70% ethanol. Samples then underwent a series of dehydration in water-ethanol, after which they were cleared with xylene and infiltrated with molten paraffin wax. Samples were then cut into 4 *µ*m-thick sections and stained with a Verhoeff’s-VanGieson elastic stain. Scanned slices (Fig. 2) were viewed with Zen 3.3 (blue edition), from which input images with calibration scale bars were generated and processed using Matlab R2020a, to obtain the respective intima-media proportion. The media was found to occupy 90 ± 3%, 76 ± 7%, and 66 ± 6% of the aortic wall thickness in the ascending, aortic arch, and descending thoracic regions, respectively (Table 3). Therefore, to represent the thickness of the intima-media in our computer models, we used seven elements in the ascending region (88% of the aortic wall), six elements in the aortic arch region (75% of the aortic wall), and five elements (63% of the aortic wall) in the descending thoracic region.

**Table 3:** Intima-media proportion measured from 4 porcine aortas

Aorta	Intima-media proportion [%]		
	Ascending	Arch	Descending thoracic
1	90	67	64
2	86	77	60
3	90	85	70
4	93	76	72
Average	90	76	66
Standard deviation	3	7	6

### Solid Matrix

#### Constitutive Modeling

Experimental second P–K membrane tension vs. Green strain curves obtained from planar biaxial tensile tests performed in another independent study on similar tissues in Ref. [18] were used to fit constitutive models to describe the solid matrix. A total of nine samples per region acquired from nine animals were used. One sample from each of the ascending and descending thoracic regions did not display appropriate stress-stretch curves and these were eliminated from the curve-fitting process. A total of nine equibiaxial and non-equibiaxial stretching protocols were used, as detailed previously in Ref. [33].

The selection of the constitutive formulation was based on equations that were readily available in FEBioStudio, that function with the Donnan law, and that yielded acceptable curve fits with the experimental stress-strain curves of the porcine thoracic aortas (details in Ref. [18]). Amongst the constitutive formulations that are widely used in modeling aortic tissue is the Holzapfel-Gasser-Odgen model [9]. However, this constitutive relationship did not yield acceptable curve fits against our experimental data despite treating the fiber directions, which were experimentally unavailable, as unknown parameters in the optimization process. Instead, a Holmes–Mow [34] constitutive law was used to model the solid matrix and is given by:

7 $$W=\frac{1}{2}c\left({e}^{Q}-1\right)$$

8 $$c=\frac{\lambda +2\mu }{2\beta }$$

9 $$Q=\frac{\beta }{\lambda +2\mu }\left[\left(2\mu -\lambda \right)\left({I}_{1}-3\right)+\lambda \left({I}_{2}-3\right)-\left(\lambda +2\mu \right)\text{ln}{J}^{2}\right]$$

where ${I}_{1}$ and ${I}_{2}$ are the first and second invariants of the right Cauchy-Green tensor, $\lambda$ and $\mu$ are the Lamé parameters, $\beta$ is an exponential stiffening coefficient, and $J$ is the determinant of the deformation gradient. Because the modeling framework consists of a fluid phase and a porous solid phase, we imposed $\lambda$ = 0 to ensure a Poisson’s ratio of zero [35]. However, given the equivalence between the response of an incompressible material and that of a biphasic material under short time loading [35], we also imposed $J$ = 1. This equivalence between a short time biphasic response and an incompressible elastic material was introduced by Ateshian et al. [35]. Briefly, in response to a sudden loading, except at the boundaries, the pores change in shape, not volume, as the interstitial fluid does not have time to leave the tissue. Under such conditions, the divergence of the fluid flux is null and the equations for the Cauchy stress tensor, conservation of mass and conservation of linear momentum become identical for both biphasic and incompressible elastic responses, as well as the boundary condition except for the pressure at the boundaries. This assumption was possible in this work, as the response of the aortic rings due to swelling of the FCD, as measured by reaching a stable opening angle, was quasi-instantaneous, as verified numerically in this work for all modelled rings, physically in our previous experimental work [19], and as previously established in similar work on rodent tissue in Ref. [26].

The equations therefore reduced to $W=\frac{\mu }{2\beta }\left({e}^{Q}-1\right)$, where $Q=\beta \left({I}_{1}-3\right)$. Therefore, two material constants, $\mu$>0 and $\beta$>0, were fitted according to the protocol outlined in Ref. [33]. Briefly, the objective function $\Vert |{T}_{11\_exp}^{S}-{T}_{11\_theo}^{S}|+|{T}_{22\_exp}^{S}-{T}_{22\_theo}^{S}|+|{T}_{12\_exp}^{S}-{T}_{12\_theo}^{S}|\Vert$ was minimized, where ${T}^{S}$ represents the 2^nd^ P–K membrane tension from experimentally ($\_exp$) and theoretically ($\_theo$) derived membrane tensions, via tensor

10 $${{\varvec{T}}}^{{\varvec{S}}}=\left[\begin{array}{ccc}\frac{\partial w}{\partial {E}_{11}}& \frac{\partial w}{\partial {E}_{12}}& 0\\ \frac{\partial w}{\partial {E}_{21}}& \frac{\partial w}{\partial {E}_{22}}& 0\\ 0& 0& 0\end{array}\right]$$

In this expression, $w=H\widehat{W}$, where H is the undeformed thickness, and $\widehat{W}$ is a reduced function of $W$ as follows. Given that the shear strains are minimal, and using the incompressibility assumption $J=\text{det}F=1$, which makes it possible to express ${E}_{33}$ in terms of ${E}_{11}$ and ${E}_{22}$, we can indeed introduce a reduced strain energy density function such that $\widehat{W}=\widehat{W}\left({E}_{11},{E}_{22}\right)=W({E}_{11},{E}_{22},{E}_{12},{E}_{33})$. This allowed us to express the Holmes–Mow constitutive model as a function of ${E}_{11}$, and ${E}_{22}$ only, such that $\widehat{W}=\frac{\mu }{2\beta }\left({e}^{Q}-1\right)$, where $Q=2\beta \left({E}_{11}+{E}_{22}+\frac{1}{2}\left[\frac{1}{\Delta }-1\right]\right)$ and $\Delta =(2{E}_{11}+1)(2{E}_{22}+1)$. Therefore, the theoretical second P–K membrane tensions were derived as:

11 $${T}_{11\_theo}^{S}=\mu \left[1-\frac{1}{\Delta \left(2{E}_{11}+1\right)}\right]{e}^{Q}$$

12 $${T}_{22\_theo}^{S}=\mu \left[1-\frac{1}{\Delta \left(2{E}_{22}+1\right)}\right]{e}^{Q}$$

13 $${T}_{12\_theo}^{S}=0$$

#### Two-Layer Material Modelling

In addition to modelling the aortic wall as one layer, we also implemented a two-layer model for the solid matrix to represent the distinct mechanical behaviors of the media-intima (inner layers) and the adventitia (outer layer) of the aorta. This was carried out in the framework developed in Ref. [33], which was used for material parameter optimization as described in “Constitutive Modeling” Sect. Consider the two intimal-medial ($M$) and adventitial ($A$) layers with undeformed thicknesses ${H}_{M}$ and ${H}_{A}$ in the reference configurations, such that $H={H}_{M}+{H}_{A}$. Herein, four positive material parameters ${\mu }_{M}$, ${\beta }_{M}$, ${\mu }_{A}$,and ${\beta }_{A}$ were considered, such that $\widehat{{W}_{M}}=\frac{{\mu }_{M}}{2{\beta }_{M}}\left({e}^{{Q}_{M}}-1\right)$ and $\widehat{{W}_{A}}=\frac{{\mu }_{A}}{2{\beta }_{A}}\left({e}^{{Q}_{A}}-1\right)$, with ${Q}_{M}=2{\beta }_{M}\left({E}_{11}+{E}_{22}+\frac{1}{2}\left[\frac{1}{\Delta }-1\right]\right)$, ${Q}_{A}=2{\beta }_{A}\left({E}_{11}+{E}_{22}+\frac{1}{2}\left[\frac{1}{\Delta }-1\right]\right)$ and $\Delta =(2{E}_{11}+1)(2{E}_{22}+1)$.

The fours constants were optimized by minimizing the objective function introduced in “Constitutive Modeling” Sect. The experimental second P–K membrane tensions were:

14 $${T}_{11\_\text{exp}\_A}^{S}=\frac{{H}_{A}}{H}{T}_{11\_\text{exp}}^{S}$$

15 $${T}_{22\_\text{exp}\_A}^{S}=\frac{{H}_{A}}{H}{T}_{{22}_{\_\text{exp}}}^{S}$$

16 $${T}_{12\_\text{exp}\_A}^{S}=\frac{{H}_{A}}{H}{T}_{12\_\text{exp}}^{S}$$

such that:

17 $${T}_{11\_\text{exp}\_A}^{S}+{T}_{11\_\text{exp}\_M}^{S}=\frac{{H}_{A}}{H}{T}_{11\_\text{exp}}^{S}+\frac{{H-H}_{A}}{H}{T}_{11\_\text{exp}}^{S}={T}_{11\_\text{exp}}^{S}$$

18 $${T}_{22\_\text{exp}\_A}^{S}+{T}_{22\_\text{exp}\_M}^{S}={T}_{{22}_{\_\text{exp}}}^{S}$$

19 $${T}_{12\_\text{exp}\_A}^{S}+{T}_{12\_\text{exp}\_M}^{S}={T}_{12\_\text{exp}}^{S}$$

The theoretical second P–K membrane tensions can be derived from the respective strain energy density functions of layers $M$ and $A$, ${w}_{M}={H}_{M}\widehat{{W}_{M}}$ and ${w}_{A}={H}_{A}\widehat{{W}_{A}}$, such that ${{\varvec{T}}}^{{\varvec{S}}}=\partial w/\partial E$, and $w={w}_{M}+{w}_{A}$. The values for ${H}_{M}$ and ${H}_{A}$ for computing the required material parameters for FEBio were calculated from the total thicknesses of the nine aortas that underwent biaxial tensile testing, which were approximately 2.00, 1.96 and 1.40 mm in the ascending, arch, and thoracic regions, respectively. Note that these thickness values are associated with the set of nine samples that underwent biaxial tests in Ref. [18], from which the material parameters for the constitutive model were obtained. These values are different from the thickness values reported in Table 1, which were obtained from the aortic rings samples tested for opening angle and GAG in Ref. [13]. Since the behaviors of the two layers may differ even if they possess equal thicknesses, a factor $r=\frac{{\mu }_{A}}{{\mu }_{M}}$ was introduced to scale the stiffness of the adventitia with respect to that of the media. The fixed relationship between ${\mu }_{A}$ and ${\mu }_{M}$ was used as a constraint during the material constant identification process. Given that conflicting results have been reported with respect to the behavior of the media and adventitia [9–11, 36–38], we explored $r$ ratio values of 0.1, 0.2, 0.5, 1, 2, 5 and 10.

#### Model Evaluation

The computer models were evaluated by comparing simulated opening angles to experimental ones measured in Ref. [19], as detailed in the results.

### Statistical Analysis

Results are reported as mean ± standard deviation. The material parameters were determined in MATLAB using nonlinear multivariate optimization, at the 95% (2-tailed) confidence interval (CI) with their Pearson correlation coefficient.

## Results

### Material Constants

#### One-Layer Matrix

The material constants for the Holmes–Mow constitutive equation, obtained from the use of all 9 stretching protocols for a one-layer solid matrix are summarized in Table 4. The Pearson’s correlation coefficients for a one-layered model were all above 0.93 in all directions and for all the aortic regions considered, reflecting an overall good match between the experimental and predicted theoretical data. Representative graphs are provided in Supplemental Fig. S1–S3.

**Table 4:** Holmes–Mow material parameters (mea*n *± standard deviation) derived from all protocols for a one-layer solid matrix

Region	Ascending	Aortic arch	Descending thoracic
$\upmu$ [N/m]	61.31 ± 1.45	65.99 ± 1.63	58.90 ± 1.46
$\upbeta$ [−]	2.09 ± 0.06	2.56 ± 0.08	2.37 ± 0.07
R^2^–FD	0.94	0.94	0.93
R^2^–XD	0.94	0.94	0.95

R^2^–FD and R^2^–XD are the Pearson correlation coefficients between the experimental and predicted membrane tensions in the fiber direction and cross-fiber directions respectively

#### Two-Layer Matrix

Material parameters for the intima-media and adventitia were also obtained using a two-layer model and are summarized in Tables 5, 6, and 7 for each of the ascending, aortic arch, and descending thoracic regions. Not all ratios $r$ between the stiffness of the adventitia and media yielded acceptable curve fits. In the ascending region (Table 5), the best curve fits between the experimental and theoretical curves were achieved when $r$ was 0.1, 0.2, and 0.5, with Pearson’s correlation coefficients 0.93 or above. The worst curve fits between experimental and theoretical curves were achieved when $r$ was 2, 5, and 10, with Pearson’s correlation coefficients in the range of 0.76–0.87. In the aortic arch region, the best curve fits were achieved when $r$ was 0.1, 0.2, 0.5 and 1, with Pearson’s correlation coefficients 0.93 or above, and the worst curve fits were observed when $r$ was 5 and 10. Finally, in the descending thoracic region, the best curve fits were achieved when $r$ was 0.2, 0.5, 1 and 2, with Pearson’s correlation coefficients 0.94 or above.

**Table 5:** Holmes–Mow material parameters derived from all protocols for the media and adventitia of a two-layer solid matrix in the ascending region, where the media occupied 88% of the aortic wall

$r$	Media		Adventitia	R^2^–FD	R^2^–XD
	${\upmu }_{\text{M}}$[N/m]	${\upbeta }_{\text{M}}$[−]	${\upbeta }_{\text{A}}$[−]		
**0.1**	**55.65 ± 1.29**	**2.00 ± 0.06**	**2.77 ± 0.08**	**0.94**	**0.94**
**0.2**	**50.88 ± 1.25**	**2.20 ± 0.06**	**1.49 ± 0.06**	**0.94**	**0.94**
**0.5**	**38.99 ± 1.24**	**2.75 ± 0.08**	**0.00 ± 0.10**	**0.93**	**0.94**
1	23.85 ± 1.23	3.85 ± 0.11	0.00 ± 0.12	0.90	0.91
2	11.92 ± 1.07	5.27 ± 0.19	0.00 ± 0.12	0.86	0.87
5	4.20 ± 0.71	7.17 ± 0.33	0.00 ± 0.10	0.80	0.80
10	1.95 ± 0.47	8.50 ± 0.46	0.00 ± 0.10	0.76	0.76

R^2^–FD and R^2^–XD are the Pearson correlation coefficients between the experimental and predicted membrane tensions in the fiber direction and cross-fiber directions respectively. ${\upmu }_{\text{A}}$ can be calculated from the relationship $\text{r}=\upmu \_\text{A}/\upmu \_\text{M}$

The material parameters for *r* ratios that generated acceptable curve fits, scored with Pearson’s correlation coefficients above 0.93, are bolded

**Table 6:** Holmes–Mow material parameters derived from all protocols for the media and adventitia of a two-layer solid matrix in the aortic arch region, where the media occupied 75% of the aortic wall

$\text{r}$	Media		Adventitia	R^2^–FD	R^2^–XD
	${\upmu }_{\text{M}}$[N/m]	${\upbeta }_{\text{M}}$[−]	${\upbeta }_{\text{A}}$[−]		
**0.1**	**57.36 ± 1.35**	**2.14 ± 0.07**	**5.40 ± 0.16**	**0.93**	**0.93**
**0.2**	**54.44 ± 1.30**	**2.28 ± 0.07**	**3.64 ± 0.10**	**0.94**	**0.94**
**0.5**	**43.30 ± 1.18**	**2.90 ± 0.08**	**1.82 ± 0.07**	**0.94**	**0.94**
**1**	**33.79 ± 1.19**	**3.49 ± 0.10**	**0.34 ± 0.11**	**0.93**	**0.94**
2	19.88 ± 1.16	4.88 ± 0.16	0.00 ± 0.12	0.91	0.92
5	7.80 ± 0.90	7.12 ± 0.28	0.00 ± 0.12	0.86	0.86
10	3.75 ± 0.66	8.71 ± 0.41	0.00 ± 0.12	0.82	0.83

R^2^–FD and R^2^–XD are the Pearson correlation coefficients between the experimental and predicted membrane tensions in the fiber direction and cross-fiber directions respectively. ${\upmu }_{\text{A}}$ can be calculated from the relationship $\text{r}=\upmu \_\text{A}/\upmu \_\text{M}$

The material parameters for *r* ratios that generated acceptable curve fits, scored with Pearson’s correlation coefficients above 0.93, are bolded

**Table 7:** Holmes–Mow material parameters derived from all protocols for the media and adventitia of a two-layer solid matrix in the descending thoracic region, where the media occupied 63% of the aortic wall

$\text{r}$	Media		Adventitia	R^2^–FD	R^2^–XD
	${\upmu }_{\text{M}}$[N/m]	${\upbeta }_{\text{M}}$[−]	${\upbeta }_{\text{A}}$[−]		
0.1	45.75 ± 1.06	1.86 ± 0.07	6.06 ± 0.21	0.90	0.93
**0.2**	**45.98 ± 1.06**	**1.85 ± 0.07**	**4.51 ± 0.13**	**0.92**	**0.94**
**0.5**	**39.33 ± 0.95**	**2.18 ± 0.07**	**2.68 ± 0.07**	**0.93**	**0.95**
**1**	**28.60 ± 0.86**	**2.98 ± 0.08**	**1.78 ± 0.07**	**0.93**	**0.95**
**2**	**19.05 ± 0.87**	**3.92 ± 0.11**	**0.98 ± 0.08**	**0.92**	**0.94**
5	8.45 ± 0.76	5.71 ± 0.20	0.62 ± 0.09	0.89	0.91
10	3.55 ± 0.54	7.47 ± 0.32	1.17 ± 0.08	0.85	0.87

R^2^–FD and R^2^–XD are the Pearson correlation coefficients between the experimental and predicted membrane tensions in the fiber direction and cross-fiber directions respectively. ${\upmu }_{\text{A}}$ can be calculated from the relationship $\text{r}=\upmu \_\text{A}/\upmu \_\text{M}$

The material parameters for *r* ratios that generated acceptable curve fits, scored with Pearson’s correlation coefficients above 0.93, are bolded

### Opening Angle

#### One-Layer Matrix

**Fig. 3:**
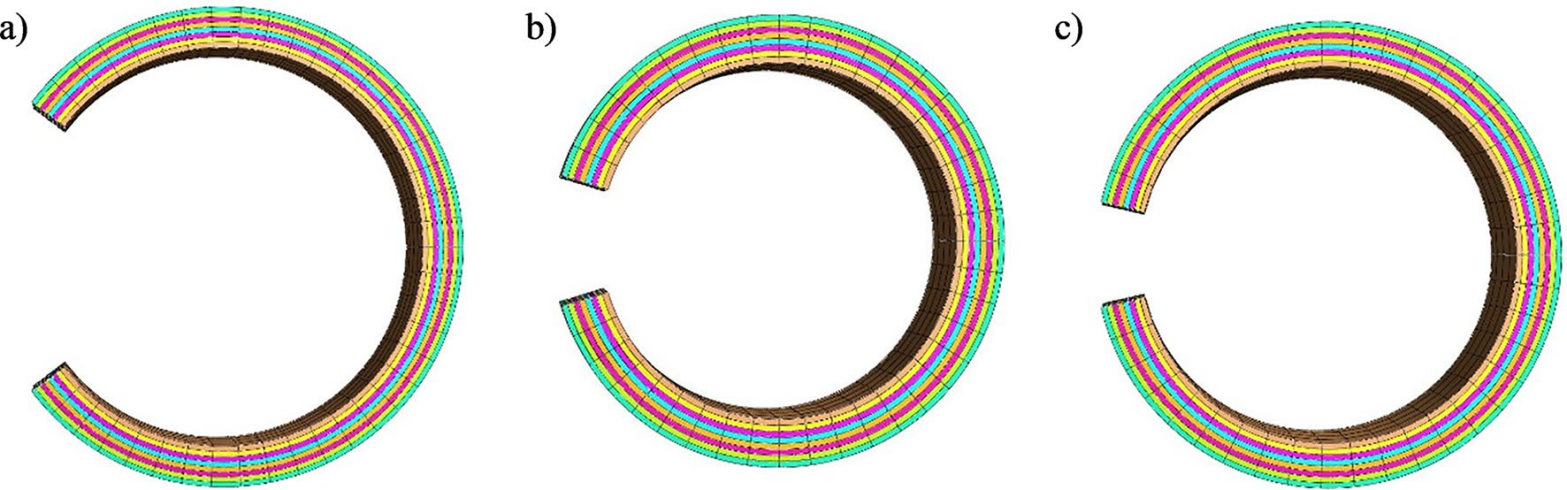
Open aortic rings (1/2 model) of the **a** ascending, **b** aortic arch, and **c** descending thoracic regions.

Open ring models for each of the ascending, aortic arch, and descending thoracic regions using one layer for the solid matrix are shown in Fig. 3, and the corresponding simulated opening angles are summarized in Table 8.

**Table 8:** Simulated opening angle results in the ascending, aortic arch, and descending thoracic regions, using a one-layer solid matrix

Region	Ascending	Aortic arch	Descending thoracic
Opening angle [degrees]	37	17	10

#### Two-Layer Matrix

The simulated opening angles in each of the ascending, arch, and descending thoracic two-layered matrix models, for each of the respective stiffness ratios $r$ that were tested are summarized in Table 9. As shown, the opening angle increases with r in all regions.

**Table 9:** Simulated opening angle results in the ascending, aortic arch, and descending thoracic regions, using a two-layer solid matrix

$\text{r}$	Opening angle [degrees]		
	Ascending	Arch	Descending thoracic
0.1	**35**	**10**	− 7
0.2	**40**	**13**	** − 1**
0.5	**54**	**21**	**8**
1	76	**28**	**16**
2	105	43	**27**
5	141	75	52
10	160	99	82

The opening angles for r ratios that generated curve fits with Pearson’s correlation coefficients above 0.93 are bolded, whereas the others are in italics

### Model Evaluation

**Fig. 4:**
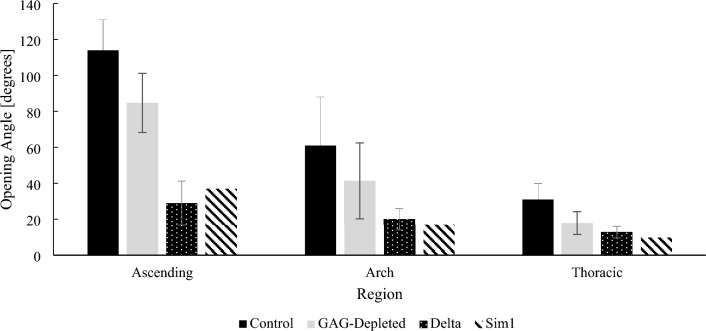
Simulated opening angles (Sim1) vs experimental opening angles from 5 control porcine aortic rings (Control) , vs experimental opening angles from 5 GAG-depleted porcine aortic rings (GAG-depleted), and vs the difference in opening angle following GAG removal (Delta) (*N *= 5 animals) [19]

The finite element results were evaluated by comparing the simulated opening angles to experimental opening angles measured in a previous study [19]. Fig. 4 summarizes the opening angle results obtained using the one-layered solid matrix with experimental results from Ref. [19]. In this previous study, we evaluated 5 sets of adjacent rings per region from 5 porcine animals. In each set, one ring was a control and did not undergo any alterations in the ECM, while the second ring was subjected to enzymatic GAG depletion, enabling us to capture the contribution of GAG to the opening angle. After GAG removal, the experimental opening angles decreased by 29 ± 12 deg, 20 ± 6 deg, and 13 ± 3 deg (denoted as “Delta” in Fig. 4) in the ascending, arch, and descending thoracic regions respectively. We believe it is the opposite of this relative decrease in the opening angle following GAG depletion that is captured by the simulation of GAG as done in the present study, and may be the best reference for comparison of our simulated results. Indeed, “Delta” represents the decrease in opening angle caused the removal of GAG from the ECM, which is therefore expected to directly reflect the effect of GAG on the opening angle. To wit, our simulated results using one-layered solid matrices (denoted as “Sim1”) demonstrated relatively small percent errors compared to the experimental GAG-associated decrease “Delta”, namely: 28, 15, and 23% in the ascending, arch and descending thoracic regions, respectively. However, the experimental opening angles of the control rings were 114 ± 17 deg, 61 ± 27 deg, 31 ± 9 deg in the ascending, arch, and descending thoracic regions, respectively (denoted as “Control” in Fig. 4) and the simulated opening angles of the one-layer models accounted for only 32%, 28%, and 32% of the experimental opening angles for control rings in Ref. [19].

Another line of interesting findings comes from the difference in simulated opening angles using a one-layered (Table 8) vs. a two-layered solid matrix (Table 9). The respective percent errors of the simulated opening angles with respect to the experimental contribution of GAG to the opening angle (“Delta”) are summarized in Table 10. Again, “Delta” is used as a reference to evaluate the simulated opening angles herein, as it reflects the experimental effect of GAG on the opening angle. In the ascending region, the smallest error of 21% was when the FCD were embedded in a two-layered solid matrix, with the intima-media 10 times stiffer than the adventitia ($r=0.1$). In the arch region, the smallest error of 5% was obtained when the solid matrix was modelled as 2 layers with the media twice stiffer than the adventitia ($r=0.5$). In the descending thoracic region, the smallest error of 23% was when the solid matrix was considered as one layer or when the intima-media and adventitia shared similar properties in a two-layer matrix ($r$ = 1), and very large errors were found when two layers were used for the solid matrix when $r$ was 2. Finally, in the descending thoracic region, although a two-layered solid matrix with $r=0.2$ provided plausible curve fits with the biaxial data, it yielded a negative opening angle of − 1°. While we have previously encountered negative opening angles computationally in [32] when a GAG profile that decreased from the intima to the adventitia was prescribed along the aortic wall, such values have not been experimentally observed in our work. As such, negative opening angles from Table 9 were excluded from the evaluation process, as they are not representative of our physical observations of opening angles for the considered animals. These observations may also suggest that $r=0.2$ is not representative of the difference in physiological mechanical response between the intima/media and adventitia.

**Table 10:** Percent errors of simulated opening angles in rings using one and two layers for the solid matrix, with respect to the experimental decrease in the opening angle caused by GAG removal “Delta”

Solid matrix number of layers	$\text{r}$	Error [%]		
		Ascending	Arch	Descending thoracic
1	–	28	15	**23**
2	0.1	**21**	50	–
2	0.2	38	35	–
2	0.5	86	**5**	38
2	1	–	40	**23**
2	2	–	–	108
2	5	–	–	–
2	10	–	–	–

The smallest errors are bolded

## Discussion

In this study, using the framework established in Ref. [26], porcine-specific aortic rings from the ascending, aortic arch, and descending thoracic regions were modelled as solid matrices with embedded FCD to simulate the effect of GAG swelling on the opening angle. The circumferential residual stress distribution was not directly quantified in this work; however, the opening angle, which is a relatively standard and practical measure of residual stress in blood vessels, was directly evaluated and used to evaluate the impact on circumferential residual stress. Parameters for ring dimensions, FCD, and the solid matrix required for the models were obtained from previous experimental testing in our laboratory of porcine thoracic aortas from animals aged 5–6 months and weighing approximately 90–100 kg. It is important to note that the FCD in these models, due to limitations in our experimental techniques, only account for the swelling effect of sulfated GAG. The FCD were prescribed in eight equally sized domains through the aortic wall. The significance of these specified FCD distributions stems from the precision of the experimental data on GAG distribution, from which FCDs were calculated. This precision was achieved through our experimental design, enabling us to quantify of GAG levels in approximately 300 µm thin slices through the thickness of the aortic wall [13]. Overall, the FCD distribution in all investigated regions of the aortic tree decreased from the inner layers to the outer layers. However, in contrast to Ref. [26], the FCD levels were not null in the outermost adventitia of the porcine thoracic aortas in our study. The presence of GAG in the adventitia has also been supported by other studies [16, 39].

The responses of the rings were evaluated in one- and two-layered solid matrices to simulate the difference in mechanical behavior between the intima-media and the adventitia of the aortic wall. The proportion of the intima-media with respect to the aortic thickness was obtained using histological sections from each of the ascending, arch, and descending thoracic regions, and was found to occupy approximately 90, 76, and 66%, respectively. These are similar to results reported by Sokolis [12] who measured the mean thickness of the intima-media at different anatomical locations of the aortic tree (cf. Fig. 5A in Ref. [12]), where, however, the intima-media occupied a smaller proportion in the ascending region compared to our animals. Specifically, the intima-media occupied approximately ~ 78% in the ascending, ~ 70–88% in the arch, and ~ 50–80% in the thoracic region, as estimated from the original figures. The mid-thoracic region in Ref. [12], in which the intima-media occupied about 70% of the aortic wall, best aligned with the descending thoracic region described in our study. The intima-media proportions were also evaluated in the thoracic region by other researchers, with Peña et al. [11] reporting that the intima and media occupy approximately 73 and 64% in the upper (end of arch to fourth intercostal artery) and lower descending thoracic regions, respectively, and Guidici and Spronck [8] reporting that the intima and media occupy around 69% of the wall thickness of the porcine thoracic aorta, which are also in line with our findings.

In addition to having different proportions in the aortic wall along the aortic tree, the intima-media and adventitia also possess different elastic properties [9–11, 36–38]. However, it remains unclear whether the adventitia is stiffer or more compliant than the intima-media, and whether differences are consistent along the aortic tree. Previous numerical calculations have been based on the assumption that the media is approximately 10 times stiffer than the adventitia [9], which is associated with experimental observations in Ref. [36–38]. Yu et al. [38] showed that the intima-media of the porcine aorta is stiffer than the adventitia by one order of a magnitude, and Xie et al. [37] found that the intima-media of blood vessels is 3 to 4 times stiffer than the adventitia. Our results are in line with these findings in that, in most cases, better curve fits were obtained when the intima-media was stiffer than the adventitia, except in the descending thoracic region, where both stiffer and softer behaviors provided satisfactory results. Specifically, our constitutive model optimization suggested that in the ascending region, the adventitia was more compliant than the intima-media, given that best curve fits were obtained when $r$ was 0.1, 0.2, and 0.5. Similar findings were obtained in the arch region, which however, also displayed good curve fits when $r$ was 1, suggesting that the adventitia could be more compliant than the intima-media, or share similar elastic properties. In the descending thoracic region, best curve fits were observed when $r$ ranged from 0.2 and 0.5 to 1 and 2, indicating that both stiffer and softer behaviors were plausible. Recent biaxial tensile testing performed by Peña et al. [11] and Amabili et al. [10] in the descending thoracic aorta of porcine and human aortas, respectively, suggested that the adventitia is stiffer than the media. Our curve fits in the descending thoracic region reflected both findings in Ref. [10, 11, 36–38], suggesting that either both behaviors of the intima-media versus the adventitia are possible in the descending thoracic region, or that more work is needed to settle this issue.

The feasibility of achieving a unique computational solution regarding the elastic behavior of the intima/media compared to the adventitia also warrants further discussion. The composite laminate plate theory tells us that multiple arrangements of different materials (e.g. different layers of reinforcement fibers oriented at different angles in an elastic matrix) may produce similar macroscopic mechanical properties [40], but when seeking to identify the material properties of blood vessels, one is after a unique solution that corresponds to the actual properties of the different constitutive layers. However, this requires a quantity of information about the layers themselves that is not typically available. As a workaround, this study explored various ratios “$r$” that scale the stiffness of the intima/media versus the adventitia, assessing them based on the quality of the curve fit between experimental and theoretical stress-strain curves obtained at the macroscopic level. The fact that multiple ratios provided a good quality curve fit (Tables 5, 6, and 7) suggested that multiple behaviors between the two layers can produce similar macroscopic mechanical biaxial properties, whereas a single solution was hoped for. Therefore, to add information to the material characterization process, we propose to use the simulation’s capability to predict the experimental contribution of GAG to the opening angle (Table 10), in conjunction with the code’s ability to provide an acceptable curve fit against experimental curves using Pearson’s correlation coefficient (Tables 5, 6, and 7). Based on this approach, and referring to Table 10, we were able to narrow solutions down to a ratio $r=0.1$ in the ascending region, as it corresponds to the smallest error between the experimental and simulated opening angles, suggesting that the intima/media is 10 times stiffer than the adventitia, again, in line with Ref. [38]. In the aortic arch and descending thoracic regions, the unique solutions would correspond to a ratio $r=0.5$, suggesting that the intima/media is 2 times stiffer than the adventitia, and to $r=1$, suggesting that the intima/media is as stiff as the adventitia, respectively. To further validate the effectiveness of computational simulation in accurately predicting the stiffness of the intima/media with respect to the adventitia, additional comparisons against experimental results would be required; however, experimental data regarding the difference in elastic properties between the layers of the aorta remain scarce and conflicting. This could be attributed to the experimental challenges associated with separating the adventitia from the media, especially in the proximal regions of the aorta due to a thin adventitia. Beyond this work, it would be generally useful to investigate the feasibility of predicting a unique solution regarding the stiffness of the intima/media compared to the adventitia without relying on opening angle results, i.e., directly in the optimization process of the biaxial data. While the stiffness ratio “$r$” could be introduced as a parameter to be optimized for, exploring other methods to predict the relative behaviors of the layers could also be useful. For example, incorporating additional known parameters to guide the optimization process, such as using known fiber directions in fiber-based models like the HGO model, could enhance the optimization process.

The opening angles simulated in both one- and two-layered solid matrices were found to decrease from the ascending to the descending thoracic region, which is in line with experimental observations [12, 13], indicating a decrease in circumferential residual stresses in the distal regions of the aorta. Because the aortic ring models only account for the Donnan swelling caused by the presence of GAG, it is expected that the simulated opening angles would better recover the experimental contribution of GAG to the opening angle, rather than the full opening angle. Indeed, we previously quantified the experimental contribution of GAG in porcine thoracic aortas as the difference in opening angle—“Delta”—between enzymatically GAG-depleted rings to controls in Ref. [19]. The errors between the simulated opening angle and experimental values of “Delta” are summarized in Table 10 and varied depending on the number of layers used for the solid matrix, as well as the stiffness ratio $r$. In the ascending, arch and descending thoracic regions, the smallest errors were 21, 5, and 23%, respectively. Herein, these errors suggest that GAG swelling are one of but not the only regulators of the opening angle, and as such circumferential residual stress, in line with our previous experimental findings [19], as detailed in the following paragraph. Unfortunately, it was not possible to report comparisons with findings from other research groups, due to the lack of literature data on this topic.

Of note, when comparing the simulated opening angle to experimental opening angles of control rings that did not undergo any ECM modifications (“Control”), large errors were found. Hence, in contrast to conclusions in [26], simulated values did not readily yield values in the range of the experimental opening angles, rather they better reproduced the experimental contribution of GAG to the opening angle, as expected. This and the minor discrepancies between the simulated opening angle and the experimental decrease caused by GAG removal suggest that GAG do not contribute to the opening angle solely through their Donnan swelling capacity, which was the only factor considered in this work. This is in line with our previous suggestions that, in addition to their fundamental swelling behavior, GAG may contribute to circumferential residual stress through their interaction with the ECM fibers [13, 19]. For instance, in addition to loss of swelling, GAG removal was also associated with a loss of circumferential prestretch in [19], a known fundamental contributor to residual stress [2]. Consistently, Mattson et al. showed that GAG also contribute to collagen fiber recruitments, which they also implemented in a constitutive modelling framework [17].

Another interesting observation in this study is the increase in opening angle with increased ratio r, suggesting that, as the intima-media becomes relatively more compliant compared to the adventitia, the opening angle increases. One reasonable explanation for this observation is that, as a material becomes more compliant, it exhibits greater flexibility and becomes less resistant to deformation. In fact, the intima-media contains higher amounts of GAG compared to the adventitia [13, 26]. Higher osmotic pressures are therefore expected to be experienced by the intima-media, and as it becomes more compliant, the deformation is amplified leading to a larger opening angle.

For the porcine samples of 5- to 6-month old animals used in our studies, the biaxial behavior of the intact samples compared to the tissue samples that underwent GAG depletion was comparable as can be determined by visual inspection from Fig. 23 in Ref. [18]. Similarly to the young porcine samples herein, no major differences in the overall biaxial behavior were detected in descending thoracic porcine aortas acquired from older animals aging 12–24 months, although indications of earlier stiffening were apparent [39]. As such, if one intends to perform a similar study using a mixture of GAG FCD in a solid matrix, care should be taken to first verify that the biaxial behavior of the tissue does not differ when GAG are removed. Indeed, for 11–15 week old female Sprague-Dawley rats, an absolute leftward shift in the mechanical stress-strain curve has been reported for samples treated with chondroitinase ABC [41].

Some study limitations need stating. The effect of hyaluronic acid, which is also present in the aortic wall, but in small proportions compared to sGAG [42], was excluded. Although the mechanical separation of the media from the adventitia has been achieved by other groups [10, 11], in our hands, despite our best efforts on the tissues considered, it was not possible to do so without arbitrarily deciding where the separation lay between both layers, which defeated the purpose of the effort. This is why scaling ratio $r$ was introduced in the model instead. In addition, given the expected minimal contribution of the intima to the mechanical behavior of the healthy aorta, the intima and media were modelled as one layer. Furthermore, the geometry of the aortic rings in this work has been simplified and modelled as a perfect cylinder. Using a more realistic geometry [7] based on animal-specific aortic rings could provide an enhanced understanding regarding the use of Donnan swelling to recover the contribution of GAG to the opening angle. Finally, while constitutive equations other than Holmes–Mow’s could have been used, such as the Holzapfel-Gasser-Ogden model among many others, it was the only one that was readily implemented in FEBioStudio for combination with Donnan swelling and afforded satisfactory curve-fits (Supplementary Figs. S1–S4).

In this work, our main goal was to recover the experimentally determined contribution of GAG to the opening angle, termed “delta”, as quantified in Ref. [19]. To achieve this, FCD were directly incorporated into the solid matrix to assess the feasibility of recovering values for “delta” computationally using the Donnan osmotic swelling. An alternative approach would have entailed the use of the multiplicative decomposition approach [7, 43] as a means to model the opening angle of the GAG-depleted aortic rings, and then the addition of FCD to test the feasibility of recovering the full opening angle of the control aortic rings.

Finally, while conventionally, the existence of residual stress in arteries was attributed to the prestretch of elastic and collagen fibers [2], the seminal work by Azeloglu et al. [26] revealed that GAG also play a role in regulating circumferential residual stress, mainly through their Donnan osmotic swelling. Building on the pioneer work of Azeloglu et al. and our pervious experimental findings [13, 19], this computational work showed that GAG contribute to circumferential residual stresses in different proportions throughout the aortic tree, with their contribution decreasing in the distal regions. To date, the majority of computational studies in the literature have focused on modelling circumferential residual stresses via either the implementation of prestretch in the aortic wall [8] or consideration of FCD [26]. The findings in this work corroborate our previous experimental studies [13, 19] and suggest that although a significant portion of the opening angle is regulated by GAG, they are not the only contributors. We therefore submit that both circumferential prestretch and swelling of GAG should be considered when modelling the opening angle in the aorta. In addition, given the coupling effect between GAG and other ECM fiber, efforts may be warranted to elucidate the interaction between the two.

## Supplementary Information

Below is the link to the electronic supplementary material.

Supplementary file1 (DOCX 1834 KB)
