# Supplementary material for: Finite Element Simulation of Opening Angle Response of Porcine Aortas Using Layer Specific GAG Distributions in One and Two Layered Solid Matrices
Source: Cardiovasc Eng Technol. 2024 Oct 2;16(1):20–33. doi: 10.1007/s13239-024-00754-x (PMC11821734; doi:10.1007/s13239-024-00754-x)
Supplement: Supplementary file 1 — Supplementary file1 (DOCX 1834 KB) [file 13239_2024_754_MOESM1_ESM.docx]

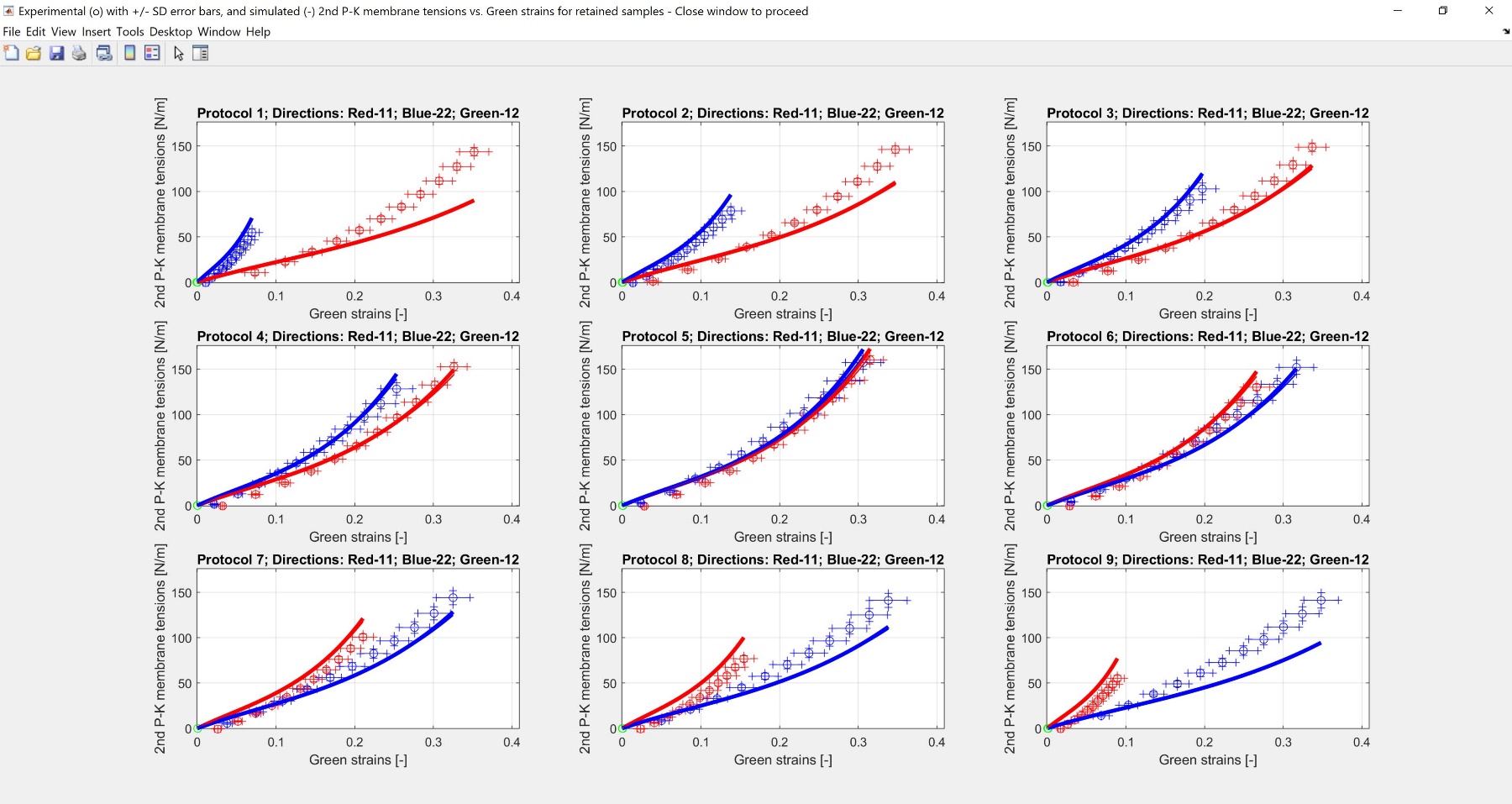


*Figure S1: Experimental data and model fit membrane tensions vs. green strains for the ascending region using one layer for the aortic wall. R^2^-FD = 0.94 and R^2^-XD = 0.94.*


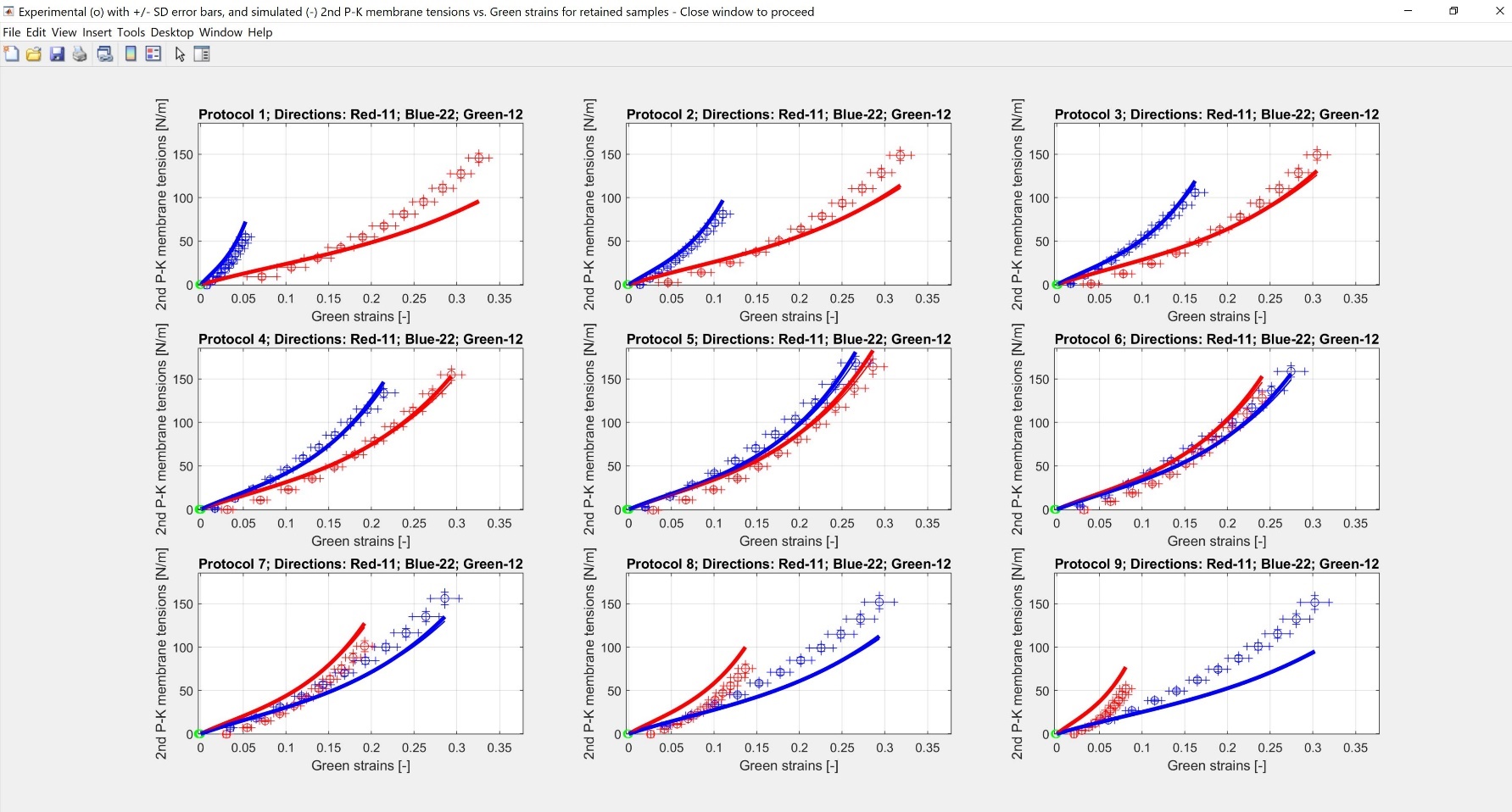


*Figure S2: Experimental data and model fit membrane tensions vs. green strains for the arch region using one layer for the aortic wall. R^2^-FD = 0.94 and R^2^-XD = 0.94.*


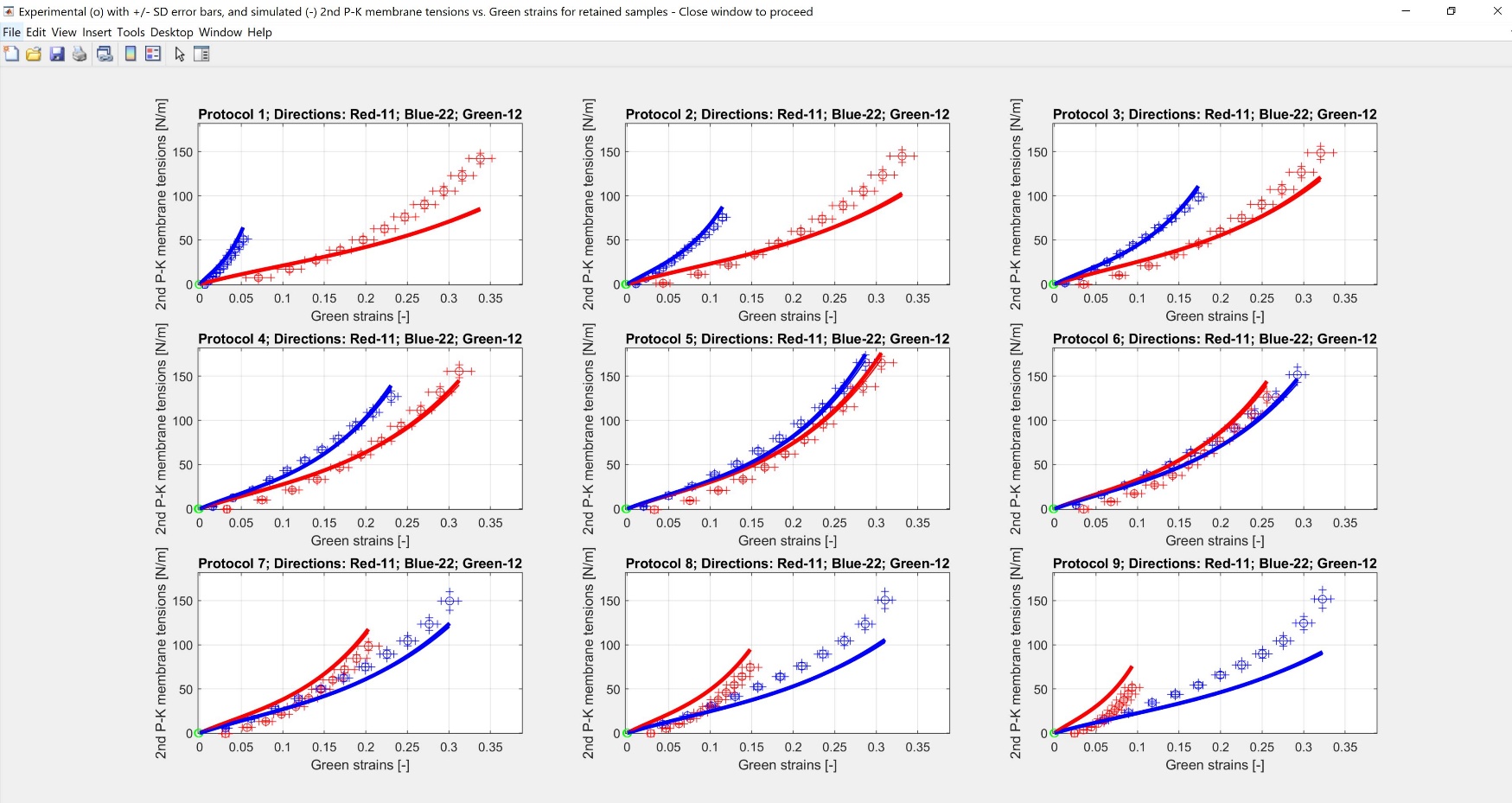


*Figure S3: Experimental data and model fit membrane tensions vs. green strains for the descending thoracic region using one layer for the aortic wall. R^2^-FD = 0.93 and R^2^-XD = 0.95.*


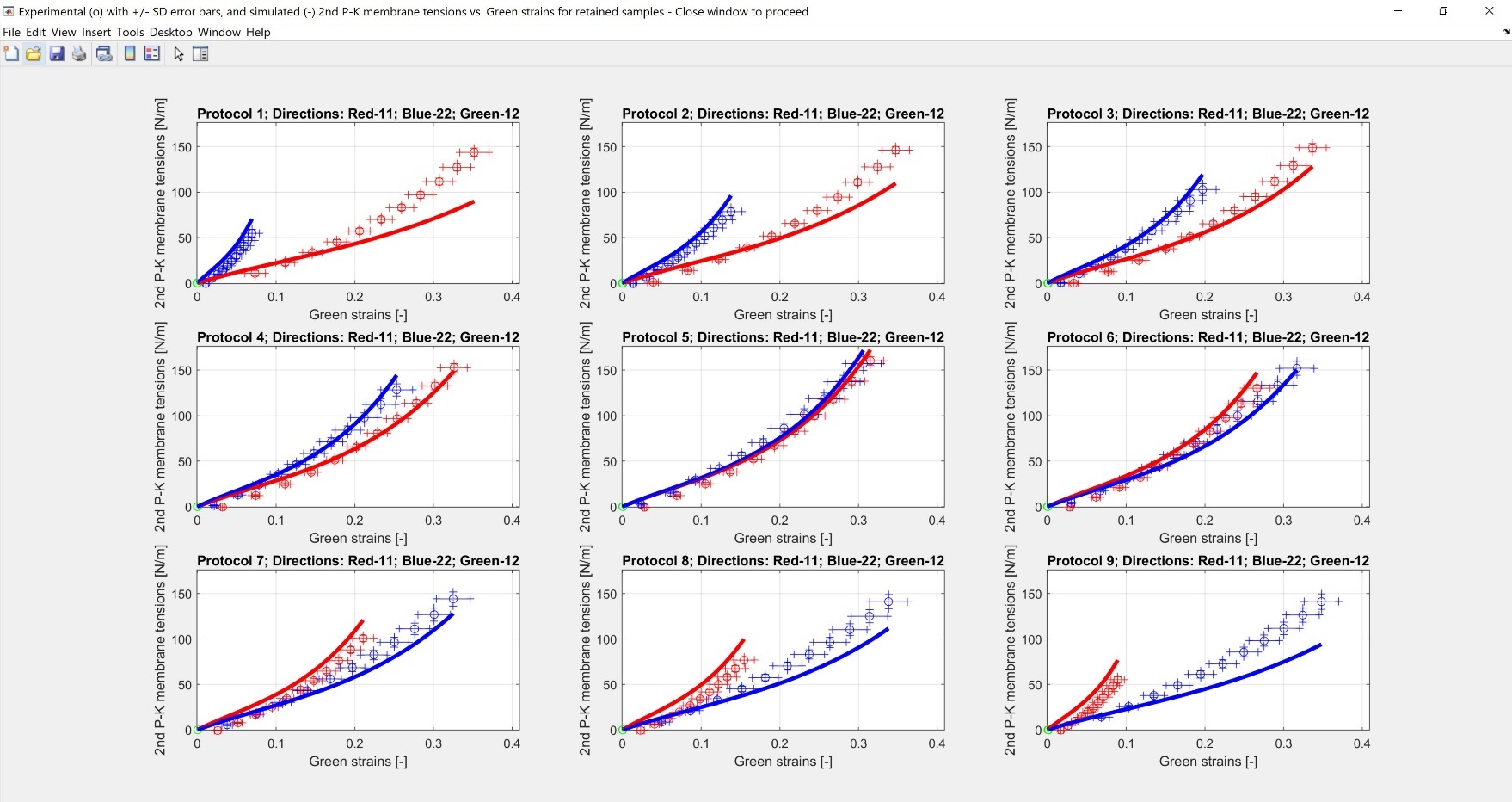


*Figure S4: Experimental data and model fit membrane tensions vs. green strains for the ascending region using two layers for the aortic wall, with* $r$ *= 0.1. R^2^-FD = 0.94 and R^2^-XD = 0.94. This is representative of curve fits that were considered acceptable for finite element modelling.*


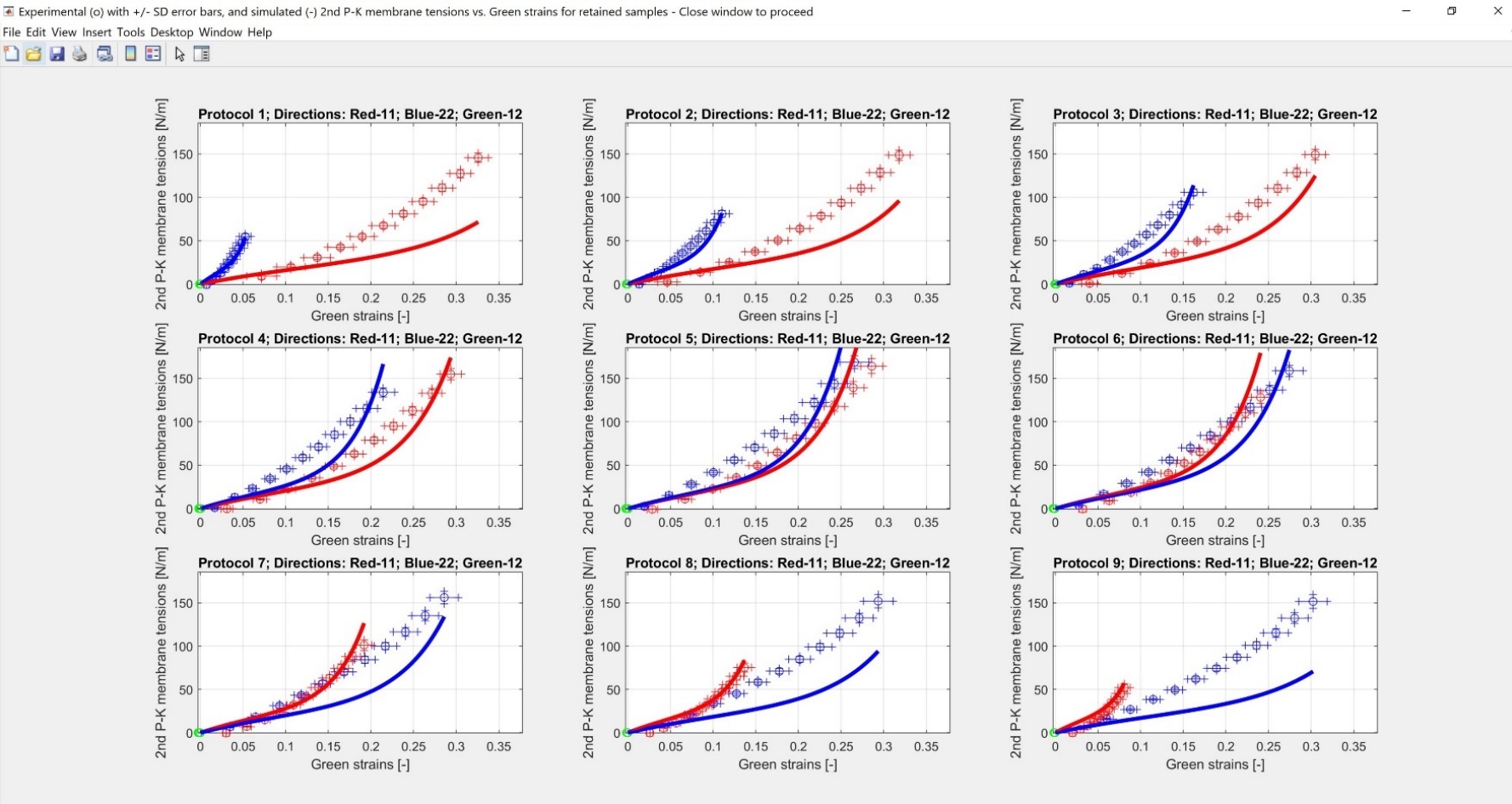


*Figure S5: Experimental data and model fit membrane tensions vs. green strains for the arch region using two layers for the aortic wall, with* $r$ *= 5. R^2^-FD = 0.86 and R^2^-XD = 0.86. This is representative of curve fits that were considered not acceptable for finite element modelling.*
